# Discovery of deoxyandrographolide and its novel effect on vascular senescence by targeting HDAC1

**DOI:** 10.1002/mco2.338

**Published:** 2023-08-17

**Authors:** Zhongxiao Lin, Hao He, Yu Xian, Jianghong Cai, Qinyang Ge, Minghao Guo, Quan Zheng, Xiaoyan Liu, Chengke Mo, Xin Zhang, Wei Qi, Youming Zhang, Lu Liang, Xi‐Yong Yu, Yi Zhun Zhu

**Affiliations:** ^1^ State Key Laboratory of Quality Research in Chinese Medicine and School of Pharmacy Macau University of Science and Technology Macau China; ^2^ Guangzhou Municipal and Guangdong Provincial Key Laboratory of Molecular Target & Clinical Pharmacology The NMPA and State Key Laboratory of Respiratory Disease School of Pharmaceutical Sciences and The Fifth Affiliated Hospital Guangzhou Medical University Guangzhou China; ^3^ Guangzhou Twelfth People's Hospital Guangzhou China; ^4^ CAS Key Laboratory of Quantitative Engineering Biology Shenzhen Institute of Synthetic Biology Shenzhen Institute of Advanced Technology Chinese Academy of Sciences Shenzhen China; ^5^ Department of Pharmacology Shanghai Key Laboratory of Bioactive Small Molecules School of Pharmacy Fudan University Shanghai China

**Keywords:** deoxyandrographolide, epigenetic, Fuzi, HDAC1, network pharmacology

## Abstract

*Aconitum carmichaelii* (Fuzi) is a traditional Chinese medicine that has been widely used in the clinic to save the dying life for over several thousand years. However, the medicinal components of Fuzi in treating vascular senescence (VS) and its potential mechanism remain unclear. In this study, a network pharmacology method was used to explore the possible components and further validated by experiments to get a candidate compound, deoxyandrographolide (DA). DA restrains aging biomarkers, such as p16, p21, γH2A.X, and p53 in vitro and in vivo blood co‐culture studies. Histone deacetylase 1 (HDAC1), mouse double minute2 (MDM2), cyclin‐dependent kinase 4, and mechanistic target of rapamycin kinase (mTOR) are predicted to be the possible targets of DA based on virtual screening. Subsequent bio‐layer interferometry results indicated that DA showed good affinity capability with HDAC1. DA enhances the protein expression of HDAC1 in the angiotensin II‐induced senescence process by inhibiting its ubiquitination degradation. Loss of HDAC1 by CRISPR/Cas9 leads to the disappearance of DA's anti‐aging property. The enhancement of HDAC1 represses H3K4me3 (a biomarker of chromosomal activity) and improves chromosome stability. RNA sequencing results also confirmed our hypothesis. Our evidence illuminated that DA may achieve as a novel compound in the treatment of VS by improving chromosome stability.

## Graphical abstract

Schematic diagram of Fuzi's active compound DA in treating vascular senescence. DA decreased aging biomarkers, such as p16, p21, p53, and γH2A.X in mRNA or protein levels. Network pharmacology identification and CRISPR‐Cas9‐V2‐based validation ulteriorly confirmed that anti‐senescence capacity of DA depended on HDAC1. DA binds to HDAC1 and inhibits its degradation by ubiquitination, which condensed the chromosomes and lowered H3K4me3 expression, and eventually decreased the expression of p21.

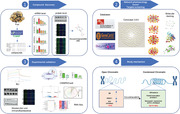

## 1 INTRODUCTION

Vascular senescence (VS) is a complex biological process (BP) that leads to vessel dysfunction and abnormal vascular morphology.^1^, ^2^ There is no simple approach to understanding the whole process of VS,^3^ and thus preventing VS‐related diseases requires long‐term attention and constant interventions. VS occurs particularly in elderly people, and its related cardiovascular diseases, such as hypertension, diabetes, and atherosclerotic disease, have become a serious global health event and public burden. Therefore, for better treatment of these VS diseases, the identification of more drug candidates is urgently needed, and traditional Chinese medicine (TCM) may provide some new options.

Fuzi is a classic TCM‐based herb, the lateral root of *Aconitum carmichaelii Debx*.^4^ The use of Fuzi in the clinic has a long tradition. The first record is in the SC East Han Dynasty (a dynasty in Chinese history, over 2000 years ago by this time), in which Fuzi was recognized to exert restorative actions that contributed to curing the syndrome of sudden yang collapse. One of its functions is helping “Yang invigoration,” meaning that it helps in saving the dying life. Although several studies have focused on rheumatoid arthritis, cardiovascular diseases, tumors, skin wounds, depression, diarrhea, gastroenteritis, and edema,^5^, ^6^, ^7^ Fuzi contains too many ingredients. Thus, its drug toxicity needs more concern and limits its widespread use. Hence, studies focused on one or more active ingredients of TCM would be more promising for exploring its clinical value. For example, aspirin derived from *willow* bark has developed into a classic anticoagulant,^8^ and paclitaxel is a natural secondary metabolite isolated from the bark of *Taxus chinensis var. mairei*, has also been transformed into a chemotherapy drug used to treat many types of cancer.^9^ In a manner of speaking, the discovery of small molecule compounds from TCM has broad prospects for the development of new drugs and therefore sparked great interest in the biology of these compounds in treating disease. However, the underlying mechanisms of the potential drugs need to be fully elucidated. With the rapid development of bioinformatics and systems biology, network pharmacology has been widely considered to be a good way to explore the mechanisms of TCM prescriptions or a single compound, which can describe the complexities among biological systems, drugs, and diseases from a network perspective.^10^, ^11^ In addition, network pharmacology integrated with experimental verification provides an optional way to find potential drug targets.

Recently, there has been accumulating evidence that epigenetic–therapeutic strategies have a crucial role in restraining the development and progression of VS diseases. Epigenetics is any factor affecting gene expression without changing the primary DNA sequence.^12^ It mainly includes DNA methylation, histone modifications, non‐coding RNA regulation, and changes in the spatial structure of chromatin.^13^ There is growing evidence points to the fact that epigenetic modification shows promising prospects in reversing phenotypic changes of aging in different cells by suppression of cellular senescence or elimination of senescent cells.^14^ For example, histone deacetylases (HDACs) remove acetyl groups from lysine residues of histones and non‐histone proteins. They modulate many cellular processes, such as chromatin remodeling and DNA repair, indicating that they may play a key role in reducing aging processes.^15^ HDAC1, a class I HDAC, maintains genomic integrity and promotes DNA double‐strand break repair through non‐homologous end‐joining (NHEJ).^16^, ^17^ The present study showed that pharmacological activation of HDAC1 promotes longevity.^18^

This paper aims to investigate whether there is an effective component in Fuzi that can regulate the VS phenotype. Subsequently, our data provided the first evidence (as far as we know) that DA significantly deceased aging biomarkers, and we were also surprised to find that DA's working mechanism is in an epigenetic‐dependent manner by targeting HDAC1 based on network pharmacology discovery and the experimental validation. We hope these findings will help to facilitate pharmacological research to reduce vascular aging processes.

## 2 RESULTS

### 2.1 Identification of potential compounds from Fuzi in treating VS

**FIGURE 1:**
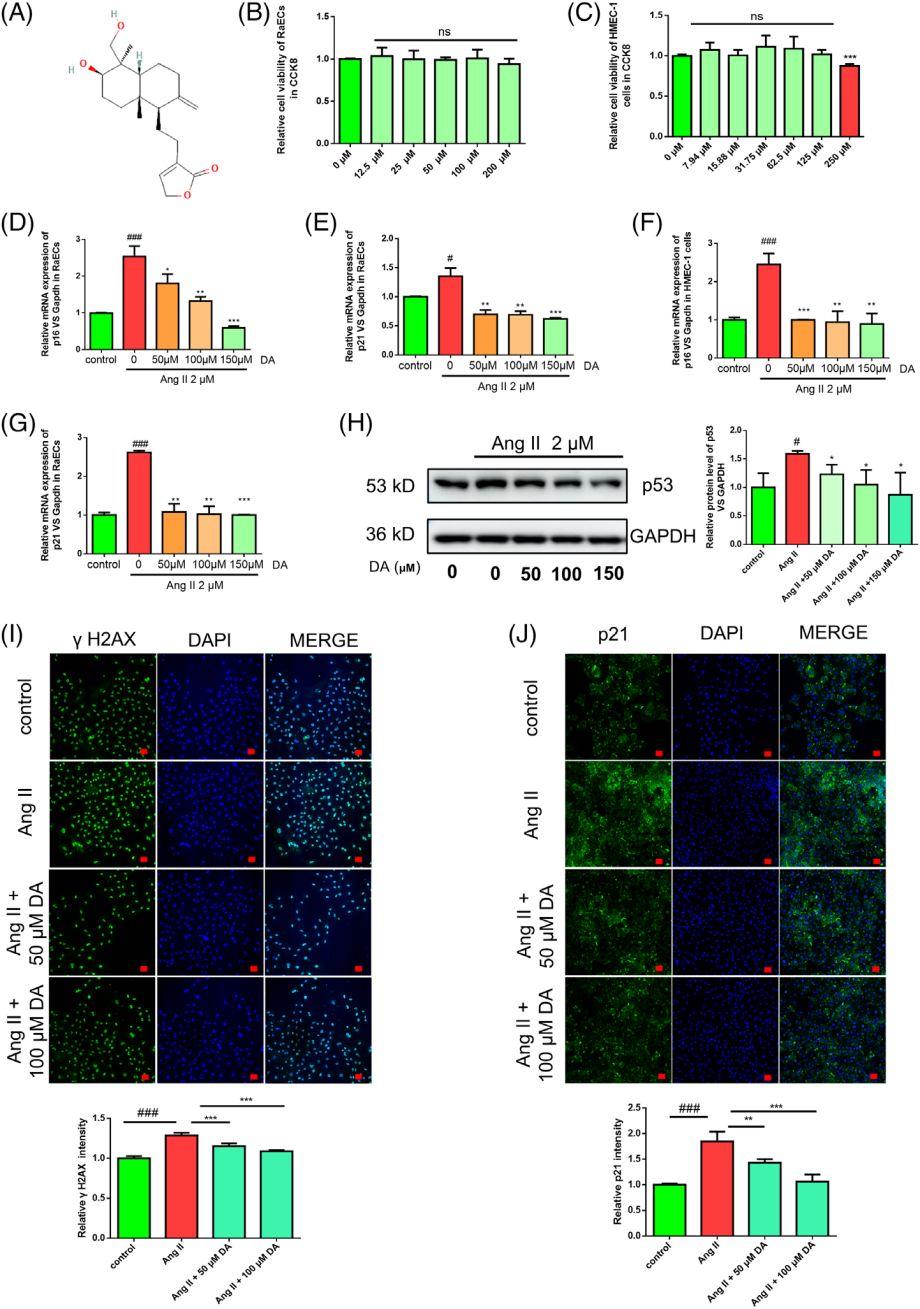
Deoxyandrographolide (DA) inhibited aging biomarkers in mRNA and protein levels in vitro. (A) The structure of DA. (B and C) Cell counting kit‐8 (CCK8) assay evaluated DA's cytotoxicity in rat aorta endothelial cells (RaECs) and human microvascular endothelial cells (HMEC‐1). (D and E) DA decreased angiotensin II (Ang II)‐induced aging biomarkers elevation of p16 and p21 in RaECs. (F and G) DA decreased Ang II‐induced aging biomarkers elevation of p16 and p21 in HMEC‐1 cells. (H) Western blot explored that DA downregulated p53. (I and J) Immunofluorescence of aging biomarkers of γH2A.X and p21 proved that DA reduced these aging biomarkers in 50 and 100 μM. Scale bar = 20 μm. Ns: non‐significant changes; ^#^ *p* < 0.05, ^##^ *p* < 0.01, ^###^ *p* < 0.001, compared with control group.^*^ *p* < 0.05, ^**^ *p* < 0.01, ^***^ *p* < 0.001, compared with model group.

A total of 65 compounds from Fuzi were collected from the Traditional Chinese Medicine Systems Pharmacology (TCMSP) database. These compounds were screened according to an oral bioavailability (OB) ≥30% and drug‐likeness (DL) ≥0.3 to obtain 10 bioactive candidates for further analysis and validation (Table 1). To assess whether these compounds were able to alleviate endothelial cell senescence, angiotensin II (Ang II) at a dose of 2 μM was used to stimulate rat aorta endothelial cells (RaECs) or human microvascular endothelial cells (HMEC‐1) for 48 h and caused a senescence phenotype.^19^ The suppression of aging biomarkers at the mRNA level or protein level is the criterion to estimate whether compounds have a positive function. Based on these settings, DA (Figure 1A) was selected as the candidate compound for the following research. The cell viability assay of DA at the 24‐h time point results in RaECs and HMEC‐1 (Figure 1B,C) showed that DA was non‐toxic under 200 μM. Thus, concentrations less than 200 μM were selected for the subsequent experiments. We exposed RaECs and HMEC‐1 cells to Ang II and found elevated levels of aging biomarkers. We selected DA concentrations of 50, 100, and 150 μM to assess its effects. Ang II significantly increased the mRNA levels of p21 and p16 in both RaECs and HMEC‐1 cells, while treatment with DA inhibited Ang II‐induced elevated expression of these genes (Figure 1D–G). We next examined the protein levels of aging biomarkers in p53, γH2A.X, and p21 in RaECs. Western blot assays showed that DA inhibited Ang II‐induced p53 expression upregulation (Figure 1H). Furthermore, the immunofluorescence results also provide evidence that DA restrained p21 and γH2A.X expression at 50 and 100 μM (Figure 1I,J). These results showed that cells treated with DA could reduce Ang II exposure‐induced elevation levels of aging biomarkers. These results suggest that DA reduced the Ang II‐induced endothelial senescent phenotype at the mRNA and protein levels.

**TABLE 1:** Active compounds from Fuzi with the filtration criteria of oral bioavailability (OB) ≥30% and drug‐likeness (DL) ≥0.3.

Mol ID	Molecule name	MW	AlogP	Hdon	Hacc	OB (%)	Caco‐2	BBB	DL	FASA−	HL
MOL002392	Deltoin	328.39	2.48	0	5	46.69	0.55	−0.12	0.37	0.32	7.7
MOL002395	Deoxyandrographolide	334.5	3.02	2	4	56.3	0.18	−0.49	0.31	0.27	2.79
MOL002397	Karakoline	377.58	−0.05	3	5	51.73	0.32	−0.03	0.73	0.15	11.1
MOL002398	Karanjin	292.3	2.94	0	4	69.56	1.22	0.62	0.34	0.26	13.15
MOL002401	Neokadsuranic acid B	452.74	7.05	1	3	43.1	0.69	−0.01	0.85	0.29	12.05
MOL002406	2,7‐Dideacetyl‐2,7‐dibenzoyl‐taxayunnanine F	776.9	3.12	1	14	39.43	−0.75	−1.3	0.38	0.38	10.95
MOL002410	Benzoylnapelline	463.67	3.12	2	5	34.06	0.19	−0.38	0.53	0.31	15.72
MOL002415	6‐Demethyldesoline	453.64	−1.97	4	8	51.87	−0.26	−0.57	0.66	0.12	13.14
MOL002422	Isotalatizidine	407.61	−0.73	3	6	50.82	−0.11	−0.62	0.73	0.14	11.59
MOL002434	Carnosifloside I_qt	456.78	5.69	2	3	38.16	0.28	−0.77	0.8	0.25	7

Abbreviations: AlogP, lipid‐water partition coefficient; BBB, blood–brain barrier permeability; Caco‐2, drug‐permeability in Caco‐2; FASA−, fractional negative accessible surface area; Hacc, hydrogen bond acceptor ability; Hdon, hydrogen bond donor ability; HL, half‐life; MW, molecular weight.

### 2.2 Network pharmacology‐based uncovering of compound–disease–target correlations and core target identification

**FIGURE 2:**
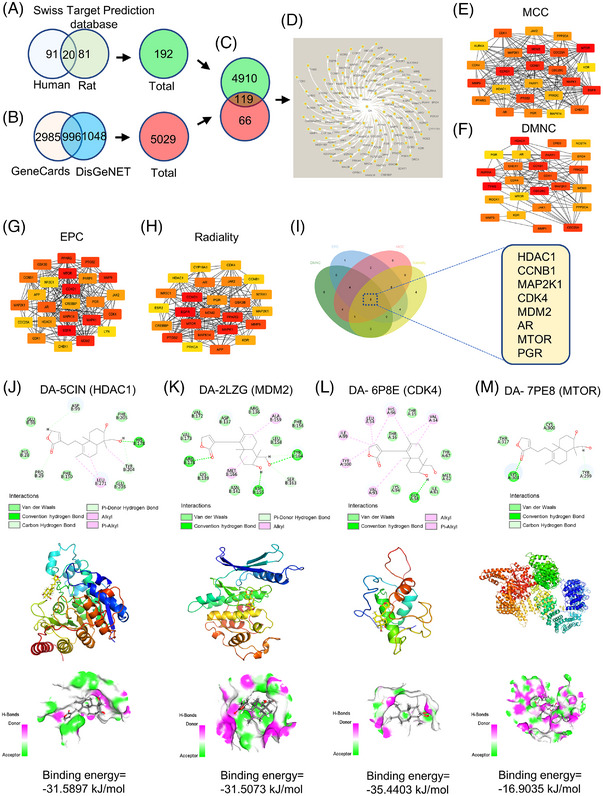
Network pharmacology‐based compound–disease–targets. (A) A total of 192 deoxyandrographolide (DA) targets predicted by the Swiss Target Prediction database. (B) A total of 5029 vascular senescence‐related targets forecasted by DisGeNET and GeneCards databases. (C) A total of 119 compound–disease–targets shown by Venn diagram. (D) Protein–protein interaction (PPI) network of 119 targets drawn by Cytoscape 3.8.0. (E–H) *MCC*, *DMNC*, *EPC*, and *Radiality* algorithms in cytoHubba tool of Cytoscape 3.8.0 software foretold the top 20 core targets. (I) Eight core targets (histone deacetylase 1 [HDAC1], cyclin B1 [CCNB1], mitogen‐activated protein kinase 1 [MAP2K1], cyclin‐dependent kinase 4 [CDK4], mouse double minute2 (MDM2), androgen receptor [AR], mechanistic target of rapamycin kinase (mTOR), and progesterone receptor [PGR]) were obtained by the intersection of four methods. (J–M) Molecular docking of HDAC1, CDK4, MDM2, and mTOR with DA. DMNC: density of maximum neighborhood component; EPC: edge percolated component; MCC: maximal clique centrality.

The network pharmacology method was employed to better identify the possible target of DA. The Swiss Target Prediction database screening results identified 111 human targets and 101 rat targets. After the deletion of duplicates, a total of 192 targets were obtained (Figure 2A). In addition, we also collected 2044 VS‐related targets from the DisGeNET database as the disease targets and 3981 genes from the GeneCards database with VS as the screening condition. After the removal of duplicates, a total of 5029 VS‐related genes were harvested (Figure 2B). The 192 active compound targets and the 5029 disease targets were used to draw a Venn diagram (Figure 2C). A total of 119 overlapping targets were obtained for subsequent research. We next performed a protein–protein interaction (PPI) network analysis of these genes; the network contained 112 nodes and 720 edges (Figure 2D), which were visualized by using Cytoscape 3.8.0 with a combined score over 0.4. To further clarify the main targets by which DA treats VS, the top 25 nodes were ranked by the *MCC*, *DMNC*, *EPC*, and *Radiality* algorithms of the cytoHubba tool in Cytoscape 3.8.0 to explore meaningful modules for targets (Figure 2E–H). cytoHubba is widely used to predict and explore key nodes and subnetworks in a given network, which has been reported before.^20^ In general, proteins with a high degree tend to be critical proteins. The Venn diagram exhibited an interaction of these four methods highlighting genes, showing that eight proteins, HDAC1, cyclin B1 (CCNB1), mitogen‐activated protein kinase kinase 1 (MAP2K1), cyclin‐dependent kinase 4 (CDK4), (MDM2), androgen receptor (AR), mechanistic target of rapamycin kinase (mTOR), and progesterone receptor (PGR), were hub targets (Figure 2I). Therefore, these targets were regarded as the potential targets of DA against VS. Subsequently, molecular docking was used to identify DA's specific binding mode and affinity to these proteins. Four of these proteins, HDAC1 (5CIN), MDM2 (2LZG), CDK4 (6P8E), and mTOR (7PE8), showed good binding affinity. As shown in Figure 2J–M, the energies of these complexes were −31.5897, −31.5073, −35.4403, and −16.9035 kJ/mol, respectively, suggesting that DA is most likely to bind closely to these proteins. In HDAC1, compound DA interacts with multiple amino acid residues in the 5CIN protein, such as ASP‐99, GLU‐98, HIS‐28, PRO‐29, PHE‐150, LEU‐271, GLU‐203, TRY‐204, and so on, capable of forming hydrogen bonds, van der Waals forces, hydrophobic, electrostatic effect, etc. The interactions of MDM2, CDK4, and mTOR are shown in Figure 2J–M. Although the results of molecular docking provided further data for the verification of the targets interacting with DA, other binding experiments are still required. In this study, we performed bio‐layer interferometry (BLI) to clarify this problem.

### 2.3 BLI‐initiated verification reveals that HDAC1 is an epigenetic target of DA

**FIGURE 3:**
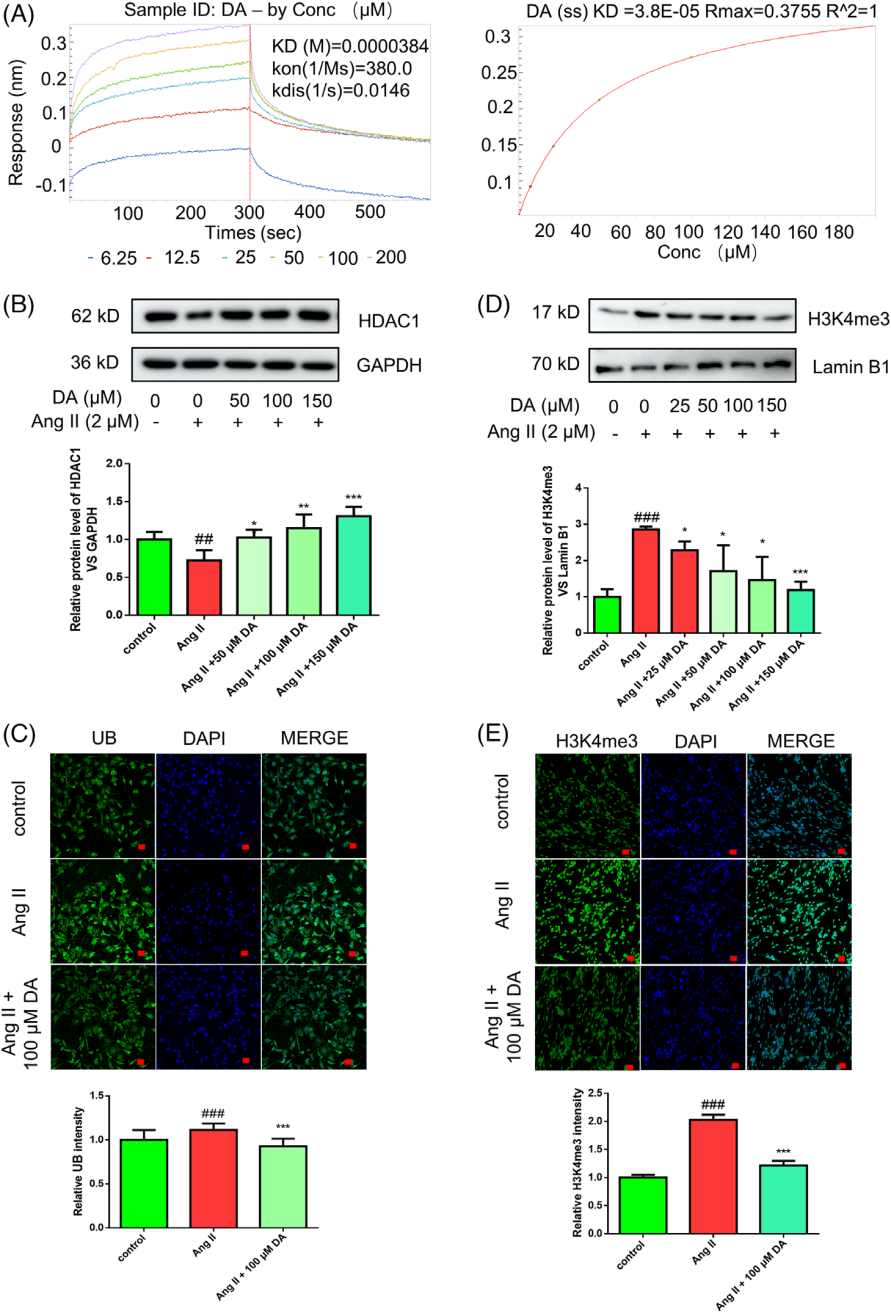
Histone deacetylase 1 (HDAC1) is a target of deoxyandrographolide (DA) in vascular senescence. (A) Bio‐layer interferometry (BLI) experiment proved that DA showed a good binding affinity with human HDAC1 protein. (B) Western blot validated that DA rescued angiotensin II (Ang II)‐induced downregulation of HDAC1 in 50, 100, and 150 μM in human microvascular endothelial cells (HMEC‐1). (C) Immunofluorescence of ubiquitin (UB) showed that DA significantly decreased Ang II‐induced UB upregulation. (D and E) Western blot and immunofluorescence confirmed that DA decreased H3K4me3 level in 50, 100, and 150 μM. Scale bar = 20 μm. ^#^ *p* < 0.05, ^##^ *p* < 0.01, ^###^ *p* < 0.001, compared with control group. ^*^ *p* < 0.05, ^**^ *p* < 0.01, ^***^ *p* < 0.001, compared with model group. Conc: concentration; Ns: non‐significant changes; Req: range equilibrium.

The HDAC1 protein is associated with DNA repair in Alzheimer's patients and healthy older adults, and pharmacological activation of HDAC1 alleviates their deleterious effects.^21^ To further verify the binding affinity of DA with HDAC1 proteins, we conducted BLI analysis to validate the binding affinity of DA (6.75, 12.5, 25, 50, 100, and 200 μM) with His‐tagged human recombinant HDAC1 immobilized onto nickel‐nitrilotriacetic acid (Ni‐NTA) probe. The results showed that DA dose‐dependently binds to HDAC1 with *K*_D_ values of 38.4 ± 1.44 μM, *k*_on_ (1/ms) of 380 ± 11.8, and *k*_dis_ (1/s) of 0.0146 ± 0.0003. Steady‐state analysis showed that the coefficient of determination (*R* ^2^) was 1, indicating that DA has a good affinity for HDAC1 (Figure 3A). To better explain how DA activated or silenced the expression of HDAC1, we generated an Ang II‐induced endothelial cell senescence model in HMEC‐1 cells. After treatment with Ang II for 48 h, we harvested total protein, and our results exhibited that the HDAC1 protein showed a downregulation tendency, which is consistent with the present report that HDAC1 is significantly downregulated in an age‐dependent manner.^22^ Interestingly, administration of DA at 50, 100, and 150 μM significantly partially restored the protein expression of total HDAC1 (Figure 3B). The enhancement of HDAC1 activity promotes DNA NHEJ repair.^23^ As DA inhibits the degradation of HDAC1, it is likely to contribute to the epigenetic homeostasis of endothelial cells. This evidence explains the reason why DA has the potential to reverse the aging phenotype. The ubiquitination degradation is a possible mechanism of HDAC1 downregulation in VS disease.^24^ Thus, we detected the total ubiquitin (UB) level. As shown in Figure 3C, the total UB level was increased after 60 h of Ang II treatment. Administration of DA decreased this elevation tendency, indicating that DA upregulated total HDAC1 by inhibiting its ubiquitination degradation. In addition, HDAC1 commonly regulates chromatin structure,^25^ condensed chromatin is relatively inactive around nucleosomes, while open chromatin is relatively loose and more active.^26^ Loss of HDAC1 usually prohibits the removal of H3K4me3 by impeding the expression of Kdm5 genes.^27^ H3K4me3 has been identified as an active chromatin marker,^28^ which is elevated in the Ang II‐induced senescence model of RaECs. Our previous chromatin immunoprecipitation (ChIp)‐seq and ChIp‐PCR data showed that the upregulation of H3K4me3‐induced p21 elevation.^19^ Pharmacological inhibition of H3K4me3 has been considered a favorable indicator to restrain VS. In this study, western blotting and immunofluorescence revealed that DA downregulated the expression of H3K4me3 in a concentration‐dependent manner (Figure 3D,E). This evidence implied that DA reverses senescence in an epigenetic manner.

### 2.4 CRISPR/Cas9‐based HDAC1 knockdown further confirmed that HDAC1 is the main target

**FIGURE 4:**
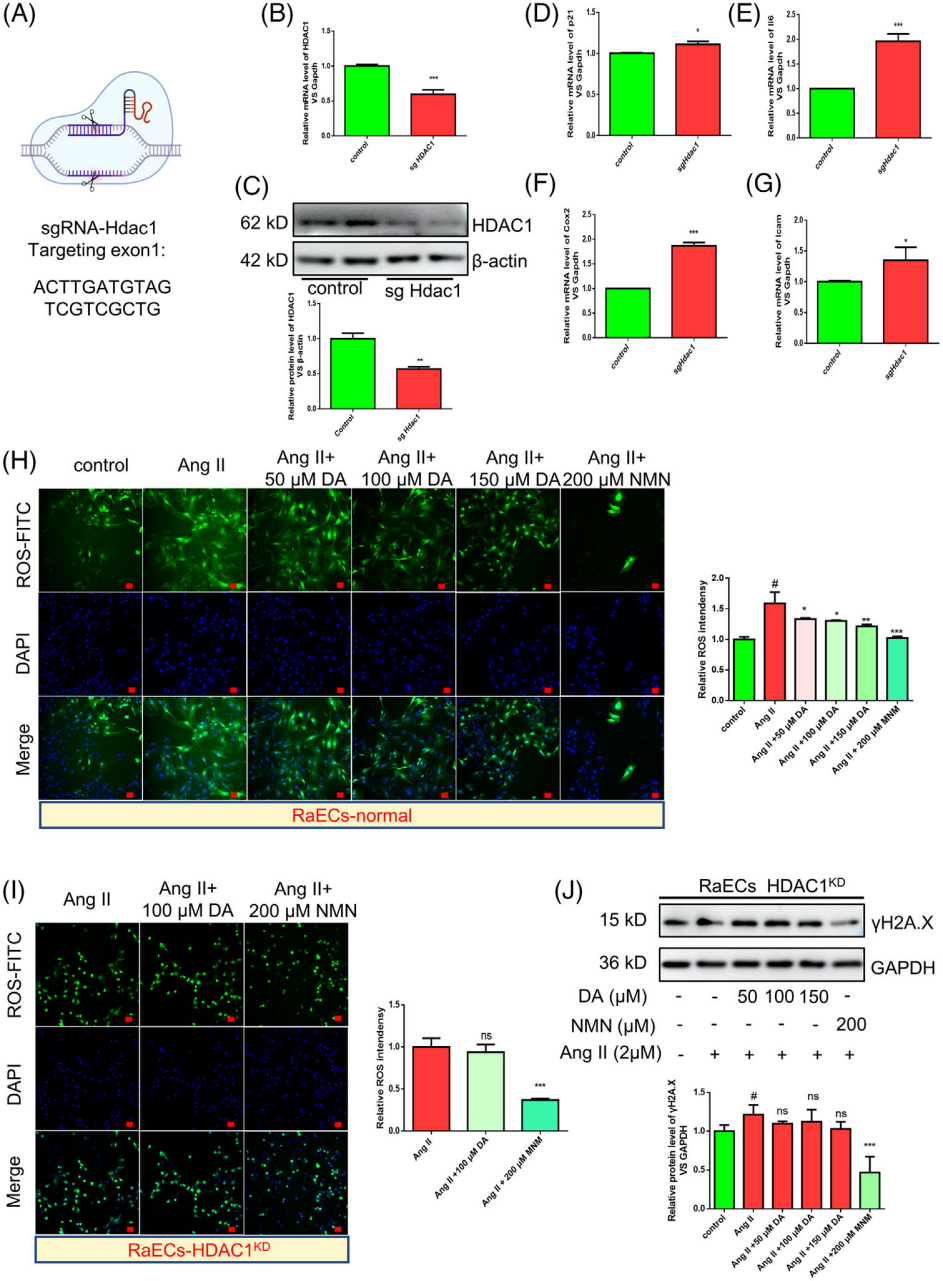
The anti‐aging processes of deoxyandrographolide (DA) depends on histone deacetylase 1 (HDAC1). (A) Schematic diagram depicting the protocol for CRISP/Cas9‐V2 system on know‐down the expression of HDAC1 (created with BioRender.com). (B) Know‐down of HDAC1 in mRNA level in rat aorta endothelial cells (RaECs). (C) Know‐down of HDAC1 in protein level in RaECs. (D–G) HDAC1 deficiency led to the increment of SASP factors, such as p21, Il6, Cox2, and Icam. (H) DA and positive control, nicotinamide mononucleotide (NMN), decreased angiotensin II (Ang II)‐induced reactive oxygen species (ROS) accumulation. (I) DA could not rescue the anti‐ROS accumulation capacity in HDAC1 know‐down RaECs, but NMN still can. (J) DA could not reverse Ang II‐induced upregulated of γH2A.X in HDAC1 know‐down RaECs, but NMN still can. Scale bar = 20 μm. ^#^ *p* < 0.05, compared with the control group. ^*^ *p* < 0.05, ^**^ *p* < 0.01, ^***^ *p* < 0.001, compared with model group. Ns: non‐significant changes.

We still questioned whether DA‐defeated vascular endothelial cell senescence depends on HDAC1. We then generated CRISPR/Cas9‐mediated HDAC1 knockdown in RaECs (Figure 4A) and 60% mRNA and 56.8% protein knockdown efficiency were examined by reverse transcription‐polymerase chain reaction (RT‐PCR) and western blotting, respectively (Figure 4B,C). To our surprise, the loss of HDAC1 led to an increment in the senescence‐associated secretory phenotype (SASP) in endothelial cells, as we observed that SASP factors (Figure 4D–G), such as p21, Il6, Cox2, and Icam, were significantly increased. In addition, HDAC1 deficiency caused the disappearance of the anti‐senescence ability of DA. From the reactive oxygen species (ROS) detection assay, DA at 50, 100, and 150 μM significantly decreased ROS accumulation (Figure 4H). However, the knockdown of HDAC1 showed no apparent change in ROS inactivation (Figure 4I). The western blot assay of total protein showed similar results in γH2A.X expression in the absence of HDAC1 (Figure 4J). However, the positive control, that is, nicotinamide mononucleotide (NMN), still played an anti‐senescence role in the Ang II‐induced model (decreased ROS and γH2A.X), for the reason that it affects many aging‐related pathways, such as the metabolic pathways of energy production in mitochondria, oxidative stress, DNA repair, chromatin remodeling, and so on.^29^, ^30^ Based on this evidence, it is clear that DA can conspicuously inhibit the aging phenotype of vascular endothelial cells based on HDAC1.

### 2.5 RNA sequencing provided comprehensive details of DA action on endothelial cell senescence

**FIGURE 5:**
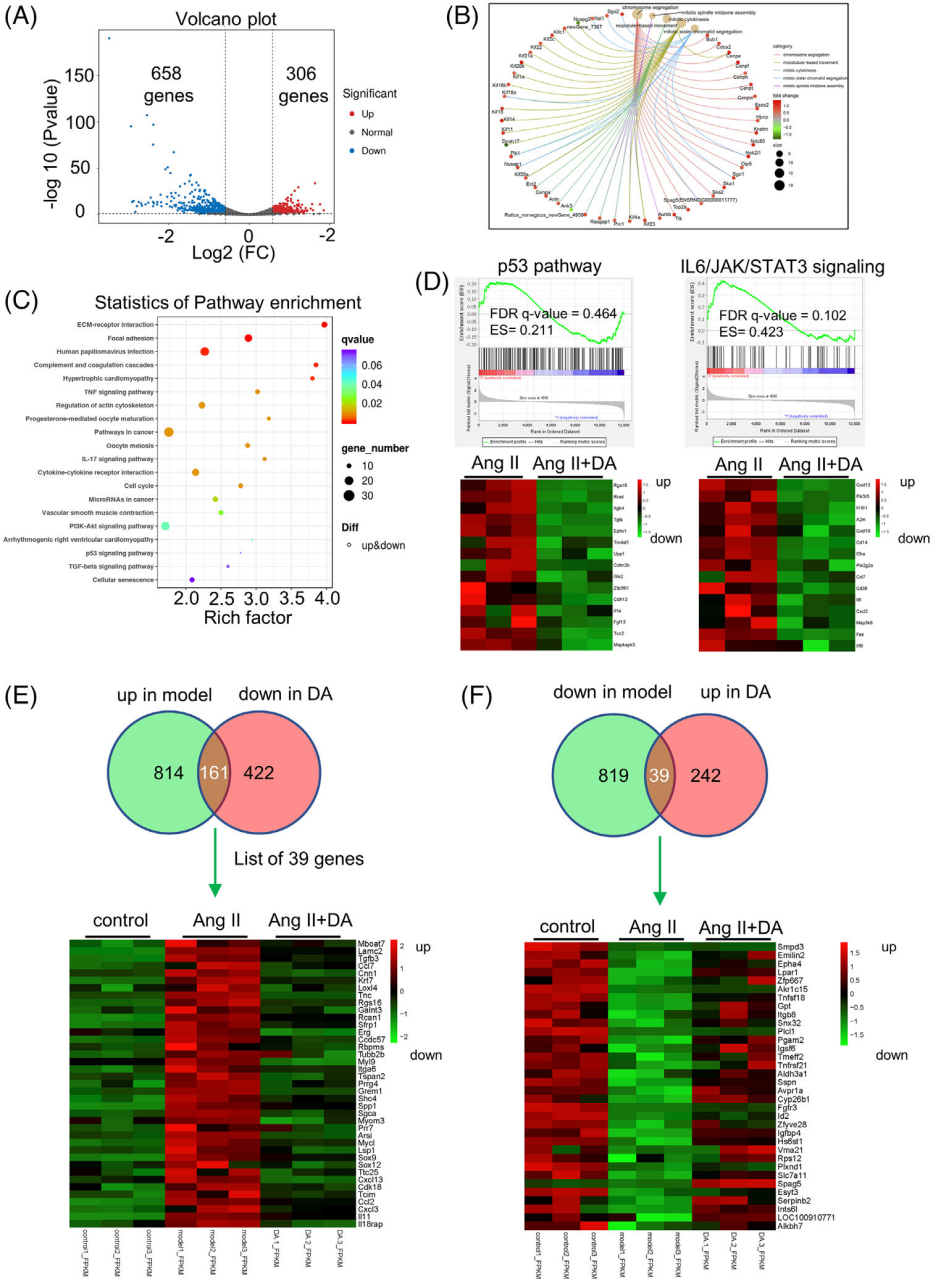
RNA sequencing provided comprehensive details of deoxyandrographolide (DA) in inhibiting endothelial cell senescence. (A) Volcano plot exhibiting the 306 upregulation and 658 downregulation genes. (B) DA‐mediated significantly changed biological processes. (C) Significantly changed Kyoto Encyclopedia of Genes and Genomes (KEGG) pathway triggered by DA. (D) Gene set enrichment analysis (GSEA) indicated that the p53 pathway and IL6/JAK/STAT3 pathway are key pathways that DA modulated. (E) Heatmap of 161 genes, which is upregulated in angiotensin II (Ang II)‐induced model and downregulated when treated with DA in 100 μM. (F) Heatmap of 39 genes, which is downregulated in Ang II‐induced model and upregulated when treated with DA in 100 μM. In heatmaps, red color indicated upregulated genes and green color indicated downregulated genes. Diff: differentially expressed genes containing.

Cellular senescence drives various pathological changes.^31^ Accordingly, further investigation into the role of DA BPs and pathways is an important field for future exploration. To provide comprehensive DA action in Ang II‐induced senescence, we conducted RNA sequencing (RNA‐seq), which is divided into a control group, Ang II group, and Ang II + DA group in RaECs. By comparing the Ang II group to Ang II + DA group in the volcano plot with a selection criterion of *p* < 0.01, log2(fold change [FC]) ≥0.5 or ≤0.5 (Figure 5A), 964 genes were identified, including 306 upregulated genes and 658 downregulated genes. The Gene Ontology (GO) method was used to analyze these differentially expressed genes (DEGs). The BP of GO is shown in Figure 5B. The top five categories of BP were chromosome segregation (*p* = 6.51 × e^−12^), mitotic spindle midzone assembly (*p* = 4.05 × e^−9^), microtubule‐based movement (*p* = 8.78 × e^−8^), mitotic cytokinesis (*p* = 1.15 × e^−08^), and mitotic sister chromatid segregation (*p* = 6.19 × e^−07^). Interestingly, HDAC1 has been well recognized to play a crucial role in chromosome segregation.^32^, ^33^ These findings echoed the sentiment that HDAC1 was a target of DA. We then analyzed the Kyoto Encyclopedia of Genes and Genomes (KEGG) pathways (Figure 5C), and the results showed that aging‐related signaling pathways, such as the p53 signaling pathway (*p* = 0.004966), cellular senescence signaling pathway (*p* = 0.005751), and longevity regulating pathway‐multiple species (*p* = 0.014608), were significantly changed. In addition, aging processes are usually accompanied by chronic inflammation.^34^ The KEGG pathways also exhibited that some inflammation pathways were modulated when treated with DA, these pathways including the tumor necrosis factor (TNF) signaling pathway (*p* = 0.000218), interleukin (IL)17 signaling pathway (*p* = 0.000450), and inflammatory mediator regulation of transient receptor potential (TRP) channels (*p* = 0.012472). We also performed gene set enrichment analysis (GSEA). As shown in Figure 5D, the senescence‐related p53 pathway (enrichment score [ES] = 0.211, false discovery rate (FDR) *q*‐value = 0.464) and inflammation‐related IL6/januskinase (JAK)/signal transducer and activator of transcription 3 (STAT3) pathway (ES = 0.423, FDR *q*‐value = 0.102) were significantly downregulated after treatment with DA at 100 μM. In these two pathways SASP factors, such as Cdkn2b, Il1β, Cxcl10, Il3, and Il6, are significantly decreased. Based on this evidence, it is clear that DA is an anti‐senescence compound. We thus analyzed which genes were significantly changed in the Ang II‐induced senescence model and DA‐treated group with the selection criteria of *p* < 0.05 and FC > 1.5 and drew them in Venn diagram and exhibited them by heatmap, as shown in Figure 5E. A total of 161 genes were upregulated in the model group but downregulated in the DA‐treated group. Among these genes, pro‐inflammatory genes (Ccl2, Ccl7) and chemokine genes (Cxcl13, Cxcl3, Il11) were upregulated when treated with Ang II but downregulated when administrated with DA. We also identified 39 genes that were downregulated in the model group but upregulated in the DA‐treated group (Figure 5F), including SLC7A11 and ID2. SLC7A11 promotes glutathione synthesis by mediating cystine uptake and glutamate release, protects cells from oxidative stress, maintains cell redox balance, and prevents cell death induced by lipid peroxidation.^35^ ID2 is highly expressed in proliferating cells but is expressed at low levels or absent in non‐proliferating cells. It is able to bind to pRB (an aging biomarker) and abolish its growth‐suppressing activity.^36^ This evidence indicated that DA was promising in reducing cellular inflammation, ferroptosis, and senescence.

### 2.6 DA downregulated aging biomarkers were observed in the co‐cultured blood of Sprague–Dawley rats

**FIGURE 6:**
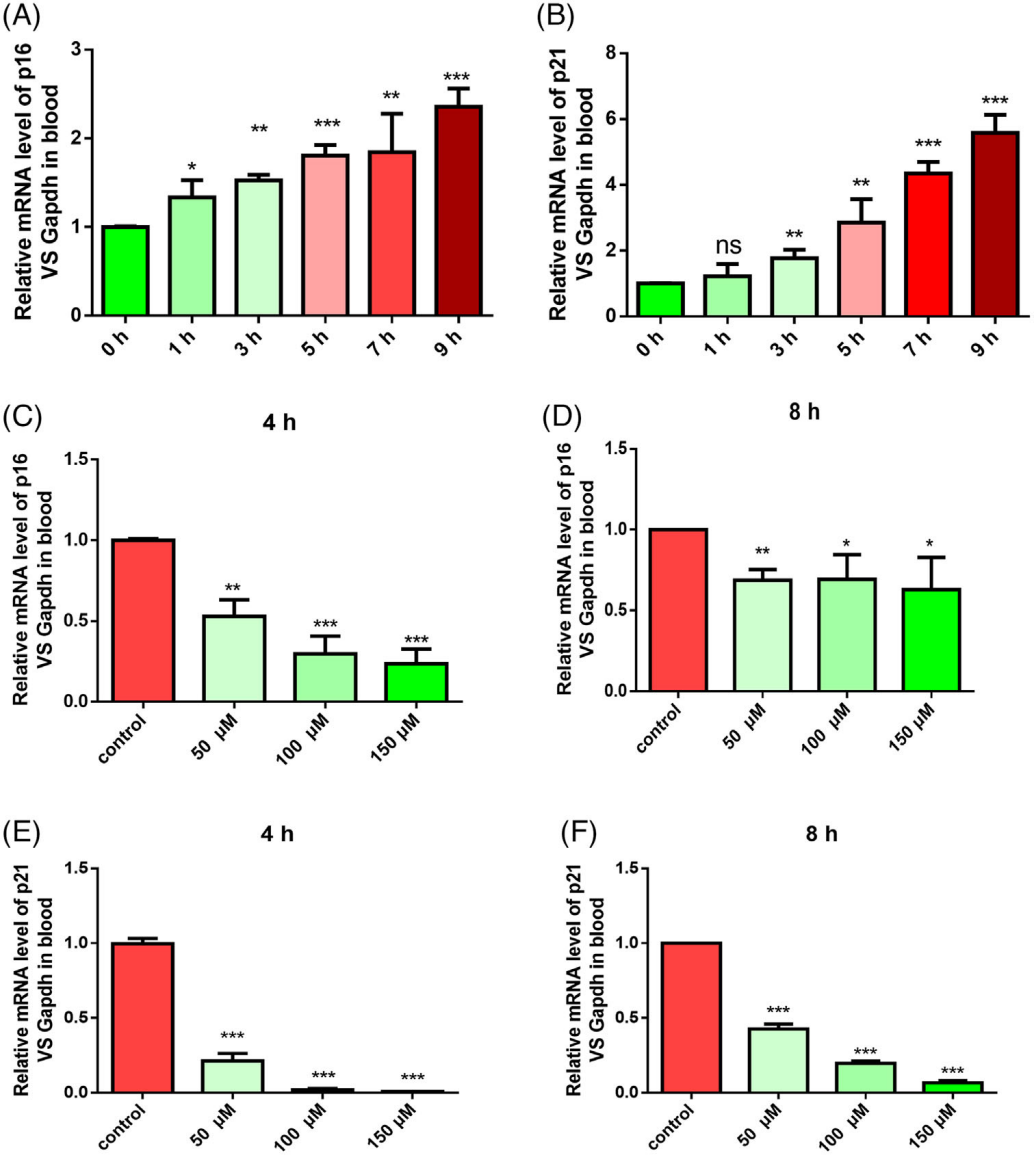
Deoxyandrographolide (DA) inhibited aging biomarkers in isolated whole blood. (A and B) Timepoint examination by reverse transcription‐polymerase chain reaction (RT‐PCR) showed that p16 and p21 were upregulated in isolated rats’ whole blood in 1, 3, 5, 7, and 9 h. (C and D) DA in 50, 100, and 150 μM significantly decreased the mRNA level of p16 in 4 and 8 h. (E and F). DA in 50, 100, and 150 μM significantly decreased mRNA level of p21 in 4 and 8 h. ^*^ *p* < 0.05, ^**^ *p* < 0.01, ^***^ *p* < 0.001, compared with the control group. Ns: non‐significant changes.

After leaving the body, cells in peripheral blood gradually become senescent or die. The mRNA level shows an elevation of aging biomarkers. Here, we used RT‐PCR to examine the expression of p16 and p21 in peripheral blood collected by an abdominal aortic puncture at time points of 0, 1, 3, 5, 7, and 9 h. As shown in Figure 6A,B, p16 and p21 expression significantly increased after the blood was isolated from bodies. Surprisingly, when whole blood was treated with DA at 50, 100, and 150 μM for 4 or 8 h, the aging biomarkers of p16 (Figure 6C,D) and p21 (Figure 6E,F) were decreased. These pieces of evidence were in line with the clinical knowledge, implying that Fuzi can be used to save dying lives would be that its active ingredient, DA, plays a crucial role in decreasing aging biomarkers.

## 3 DISCUSSION

Fuzi uses in reasonable doses and time has been shown to be of clinical value in saving dying lives in Chinese history.^37^ However, although several published studies have shown that the aqueous extract of Fuzi is involved in the generation and removal of oxygen radicals and reduced apoptosis in Wistar rats and SH‐SY5Y cells, respectively,^7^ why Fuzi is related to the therapeutic effects of vascular aging diseases is still unknown, especially which compound of Fuzi plays a crucial role in vascular aging has not been reported. Moreover, due to the complex chemical components, it remains challenging to elucidate the precise pharmacological mechanisms of Fuzi in treating VS.

In this study, we conducted systemic research using a combination of network pharmacology and experimental verification to clarify Fuzi's bioactive compound and its possible therapeutic mechanism against senescence. We first used OB and DL to screen 10 possible compounds and surveyed their functions. Some of these showed favorable effects on the cardiovascular system, such as karanjin showing antihyperglycemic activity,^38^ and isotalatizidine from Sini decoction, which may play a role against myocardial ischemia.^39^ However, whether they could suppress aging biomarkers in endothelial cells has not been reported. Karakoline and benzoylnapelline exhibit significant toxicity, so they have little value in the development as anti‐aging drugs.^40^, ^41^ Subsequently, we noticed that DA efficiency decreased the aging biomarkers of p16 and p21, and the same tendency was also observed in the protein level of aging biomarkers (γH2A.X, p21, and p53). This evidence drove us to pay more attention to this compound. To better illuminate which targets DA combined, we performed network pharmacology to identify the core target proteins of DA for treating vascular senescence. HDAC1, CCNB1, MAP2K1, CDK4, MDM2, AR, mTOR, and PGR were deemed to be hub genes. Surprisingly, these genes are highly associated with the cell cycle, sex hormones, and epigenetics. For example, the cell cycle‐related genes: CCNB1, MAP2K1, CDK4, and MDM2 proto‐oncogene; sex hormone‐related genes: AR and PGR; HDAC1 is an epigenetic‐related gene that shows the capacity to induce local condensation of chromatin and is generally considered repressors of transcription,^42^ and its role in repairing the aging DNA has been widely reported.^2^, ^21^ Rapalogs and mTOR inhibitors serve as anti‐aging therapeutics.^43^ Among these targets, our compound‐protein molecular docking and BLI data showed that DA protects against Ang II‐mediated endothelial cell senescence by directly binding to the HDAC1 protein. This binding capability caused the increased expression of HDAC1 in Ang II‐stimulated endothelial cells. To explain why DA rescued total HDAC1 protein expression in the senescence model, we investigated whether the degradation of HDAC1 protein by ubiquitination occurred in aging model based on our mRNA detection, which was not obviously changed. We then performed immunofluorescence to determine the total UB level, and the results showed that the total UB level was increased in the Ang II‐induced senescence model, which is consistent with the current study showing that the older proteomes showed increased modification by ubiquitylation.^44^ Interestingly, treatment with DA inhibited the elevation of UB levels. These results indicated that inhibition of UB levels could be the possible upstream mechanism explaining DA rescued HDAC1 degradation. Furthermore, H3K4me3 (transcriptional enhancer) has been identified as an active chromatin biomarker^45^ and its expression is usually negatively correlated with HDAC1.^46^, ^47^ Our previous study demonstrated that H3K4me3 binds to the promoters of Cdkn1a (coding for p21) in endothelial cells and human tissues of vascular disease, causing elevated p21.^19^ In this study, we observed that administration of DA can significantly downregulate the expression of H3K4me3. This finding revealed that DA reduces VS by restoring the expression of HDAC1 in senescent cells, which in turn suppresses the H3K4me3 level and decreases p21 expression. This may be an important pathway in explaining why DA has the capacity to reverse the senescent phenotype.

To further confirm whether DA reduced the expression of aging biomarkers in a manner dependent on HDAC1, we generated the lenti‐CRISPR‐Cas9 v2 method to knockdown the expression of HDAC1 in RaECs. By using puromycin screening after CRISPR/Cas9 editing, we achieved stable HDAC1‐deficient cells. These deficient cells were further stimulated with Ang II and DA for 48 h simultaneously, with the NMN as the positive control. Interestingly, we found that loss of HDAC1 led to an increment of SASP factors (p21, Il6, Cox2, and Icam) and the disappearance of the anti‐senescence effects of DA, and the NMN still showed the ability to reduce the expression of the aging biomarker‐γH2A.X. Based on this evidence, it is clear that the anti‐senescence effects of DA required HDAC1. We next performed RNA‐seq to provide a comprehensive understanding of DA's action in the Ang II‐induced model. The BPs and possible signaling pathways are shown in Figure 6B. We observed that the greatest changes in BPs were cell nuclear alterations, such as chromosome segregation and cell division‐related processes. As senescent cells usually show a state of cell cycle arrest and a slowdown in cell division,^48^ DA activates these processes, explaining why DA has anti‐aging activity. Consistent with the BP results, the KEGG pathway analysis and GSEA also provided corresponding evidence that the p53 signaling pathway, cellular senescence signaling pathway, and longevity regulating pathway were significantly changed by comparing the Ang II‐treated and untreated groups. In addition, chronic low‐grade inflammation is a common feature of the aging phenotype.^49^ DA also exhibited an anti‐inflammation response by regulating the TNF signaling pathway, IL17 signaling pathway, and inflammatory mediator regulation of TRP channels. To better illustrate the role of DA in vivo, we performed whole‐blood co‐culture testing. Astonishingly, we found that the aging markers p16 and p21 in separated whole blood were gradually upregulated, but the upregulation trend was significantly inhibited after treatment with DA. In conclusion, these results proved that DA could reduce aging markers both in vitro and in vivo. However, several questions still require further research. One potential concern is that by using network pharmacology, the possible target HDAC1 was confirmed; however, DA may also exert its effects through synergistic interaction with other targets. Second, further studies on animal and human subjects are warranted to better assess the effects of DA on managing VS.

Collectively, these preliminary studies were the first to provide a comprehensive analysis of the potential compounds, targets, and pathways of Fuzi in treating VS diseases. We demonstrated that DA decreased aging biomarkers in vitro and in vivo. DA showed good binding affinity with HDAC1 protein and thus inhibited its degradation by ubiquitination. The increment of HDAC1 decreased the H3K4me3 level and reduced the p21 expression, helping to rescue the aging phenotype. Based on the evidence mentioned above, DA can be used as a valuable lead for new therapeutics in the treatment of Ang II‐associated endothelial senescence.

## 4 MATERIALS AND METHODS

### 4.1 Reagents and drugs

P21(AB109199) was purchased from Abcam; H3K4me3 (AF5704), p21(AF5252), p53 (AF0255), UB (AF0306), and phospho‐histone H2AX (Ser139) (AF5836) antibodies were purchased from Shanghai Beyotime Biotechnology; HDAC1 (D5C6U), goat anti‐mouse secondary antibodies, goat anti‐rabbit secondary antibodies, and immunofluorescence fluorescent secondary antibodies were purchased from Cell Signaling Technology. DA was purchased from MedChemExpress (https://www.medchemexpress.cn/).

### 4.2 Animals

Male Sprague–Dawley (SD) rats (6−8 weeks old) were purchased from the Laboratory Animal Services Center of the Chinese University of Hong Kong (Hong Kong, China). All rats were housed under standard laboratory conditions. All surgeries were performed under pentobarbital (2%, dissolved in saline, 3 mL/kg) anesthesia to minimize suffering in accordance with the guidelines for the care and use of laboratory animals.

### 4.3 Screening of active compounds from Fuzi and its anti‐VS targets

OB ≥30% and DL ≥0.3 were used as the filtration criteria to obtain anti‐senescence compounds in TCMSP.^50^ Among these compounds, we found that DA attenuated senescence in RaECs and HMEC‐1 cells.

### 4.4 RaEC isolation, cell culture, and in vitro senescence model establishment

RaECs were isolated from the thoracic aortae of SD rats from 2 to 3 months old. RaECs were cultured in endothelial culture medium (Sciencell) plus 5% fetal bovine serum (FBS) and 1% endothelial cell growth supplement in the first three generations and were grown in Dulbecco's modified Eagle's medium (DMEM) with 20% FBS and 1% penicillin/streptomycin (Gibco) after the third generation. Cells were maintained at 37°C and 5% CO_2_ in a humidified atmosphere incubator. Ang II (2 μM) was used to induce VS in vitro, as reported previously.^51^ In brief, ECs were cultured in six‐well plates at a density of 1 × 10^5^ cells per well for overnight. After being washed with phosphate buffer solution (PBS) three times, the cells were incubated in a serum‐free DMEM to induce synchronization and divided into a control group, Ang II group, Ang II + DA group, and NMN group. The culture medium, Ang II, DA, and NMN were replaced every 24 h. After 48 h of stimulation, cells were harvested for subsequent experiments.

### 4.5 In vitro cytotoxicity assay

RaECs or HMEC‐1 cell lines were seeded into 96‐well plates at a density of 1 × 10^4^ cells per well (Corning) under the above conditions for 24 h prior to the experiment. The culture medium was replaced with 100 μL of DA solutions at different concentrations. Each concentration was replicated in five wells. The cells were incubated for another 24 h. PBS was used to clean each hole and then replaced it with freshly prepared serum‐free medium with 10% cell counting kit‐8 (Beyotime), and the cells were incubated for another 1–4 h. The optical density readings were performed using a multimode plate reader (Bioreader) at a wavelength of 450 nm. The absorbance was read relative to the blank well. Cell viability (%) in each well was calculated by (OD450 test – OD450 blank)/(OD450 control – OD450 blank) × 100%. A non‐toxic dose was used to evaluate the effects of DA on RaECs or HMEC‐1 cells.

### 4.6 RT‐PCR

Total RNA was isolated by TRIzol Reagent (Invitrogen). Approximately 1 μg of total RNA from each sample was reverse transcribed into cDNA and amplified using a HiFiScript gDNA removal cDNA synthesis kit (CWBIO) according to the manufacturer's instructions. PCR was then conducted using UltraSYBR Mixture (CWBIO). The primer sequences are listed in Table 2.

**TABLE 2:** Reverse transcription‐polymerase chain reaction (RT‐PCR) primers.

Primer name	Sequence (5′−3′)
Rat	
Hdac1‐F	ACTAGATAGGGACCAGCGCA
Hdac1‐R	AGCTCCTAAGCAGGCACTTG
Gapdh‐F	TTCAACGGCACAGTCAAGG
Gapdh‐R	CACCAGTGGATGCAGGGAT
p16(Cdkn2a)‐F	GTCAAAGTGGCAGCTCTCCT
p16(Cdkn2a)‐R	GATACCGCAAATACCGCACG
p21(Cdkn1a)‐F	GTGGACAGTGAGCAGTTGAG
p21(Cdkn1a)‐R	TCAGGTAGATCTTGGGC AGC
Human	
HDAC1‐F	GCTAAAGTATCACCAGAGGGT
HDAC1‐R	TGGCCTCATAGGACTCGTCA
GAPDH‐F	ACAACTTTGGTATCGTGGAAGG
GAPDH‐R	GCCATCACGCC ACAGTTTC
p16(CDKN2A)‐F	GATCCAGGTGGGTAGAAGGTC
p16(CDKN2A)‐R	CCCCTGCAAACTTCGTCCT
p21(CDKN1A)‐F	TGTCCGTCAGAACCCATGC
p21(CDKN1A)‐R	AAAGTCGAA GTTCCATCGCTC

### 4.7 Western blot

Cells were lysed in ice‐cold radioimmunoprecipitation assay (RIPA) buffer, supplemented with 1 mM phenylmethylsulfonyl fluoride. Proteins were obtained by centrifugation at 12,000 rpm for 15 min at 4°C. Equal amounts of proteins (10 μg) were loaded onto a 10%−15% sodium dodecyl sulphate‐polyacrylamide gel electrophoresis (SDS‐PAGE) unit and transferred to a nitrocellulose (NC) membrane (Millipore) by electroblotting. The NC membranes were blocked with 5% nonfat‐dried milk in Tris‐buffered saline/Tween 20 (TBST), stained with primary antibodies at dilutions of 1:1000 and then incubated overnight at 4°C. Membranes were then probed with peroxidase‐conjugated secondary antibody at a dilution of 1:10,000 (CST). The antigen–antibody complexes were then detected with enhanced chemiluminescence reagent (Beyotime), visualized using an AMERSHAmersham Image Quant 800 system (GE), and analyzed using ImageJ Software.

### 4.8 Immunofluorescence staining

RaECs were seeded on 96‐well plates overnight and then treated with Ang II and DA. After 48 h of treatment, cells were fixed with 2% paraformaldehyde for 15 min, followed by permeabilization with 0.2% Triton X‐100 in PBS for 20 min. Next, RaECs were blocked in 1% bovine serum albumin in TBST for 1 h and incubated overnight with primary antibodies (γH2A.X, p21, H3K4me3, and UB) at 4°C. Appropriate secondary antibodies were added and incubated for 2 h at room temperature. The nuclei were stained with 4,6‐diamino‐2‐phenyl indole (DAPI) for 1 h. Images were captured using an In Cell Analyzer 6000 (GE).

### 4.9 Analysis and validation of potential targets

The Swiss Target Prediction database^52^ was used to predict pharmacological targets of DA, in which rat targets and Homo sapiens targets were included. GeneCards were used to screen pathological targets of VS.^53^ Atherosclerosis (one kind of VS disease) was used as a filter to obtain gene–disease associations in DisGeNET.^54^ All primary targets of DA and pathological targets were evaluated using Venn diagrams to identify potential targets for DA against VS. The network was visualized by using Cytoscape software version 3.8.0.

### 4.10 Screening of hub genes of DA against VS and construction of an interrelated network

The STRING website (https://www.string‐db.org/) and Cytoscape software (topology parameters of Network Analyzer) 3.8.0 were used to map targets of DA action against VS and to harvest a target‐to‐target function‐related protein network, PPI. Moreover, the best targets were selected based on degree values.^20^

### 4.11 Core target and its validation

The hub genes gained above were selected for the next evaluation. Briefly, the chemical structure of DA was downloaded from PubChem (https://pubchem.ncbi.nlm.nih.gov/), and the 3D structures of the core targets were obtained from the Protein Data Bank (PDB) in PDB format. MOE version 2018.0101 was used to search and define the rotatable bonds of DA after removing water molecules, hydrogen molecules, and charges from the protein structures. The binding activity between DA and the targets was evaluated on the basis of the magnitude of the binding energy. The selected target was further confirmed by BLI assay using Octet RED96 (ForteBio). In brief, a shake speed of 1000 rpm and plate temperature of 30°C were applied to all runs. HDAC1‐His‐tag protein (50 μg/mL) was loaded on Octet Ni‐NTA biosensors (Sartorius). The background binding control used the same sensors incubated in PBS kinetics buffer without proteins. Each well containing a total volume of 200 μL DA was diluted with PBS and then added to a black polypropylene 96‐well microplate (Greiner Bio‐one) with PBS filling the rest of the wells. After washing (60 s) and the baseline step with PBS containing 2% dimethyl sulfoxide (DMSO), biosensors were immersed in wells containing different concentrations of DA to associate drugs (120–180 s). A dissociation step was then performed (120–180 s). All the data were analyzed by Octet data analysis software version 9.0. *K*_D_, *K*_on_, *K*_off_, and *R* ^2^ values were reported as the evaluation index.

### 4.12 CRISPR v2 system‐based HDAC1 knockdown in RaECs or BcECs

LentiCRISPR v2 (Plasmid #52961) was purchased from Tsingke Biotechnology Co., Ltd. The targeting Hdac1 sgRNA (sgHdac1) sequence was: ACTTGATGTAGTCGTCGCTG and the sgHdac1 plasmid was constructed according to the protocol.^55^ Then, the lentivirus was packaged with PMD2.G and PSPAX.2 using Lipofectamine 2000 transfection reagent (Thermo Fisher Scientific) in HEK 293T cells. Stable HDAC1 knockdown cells (RaECs and BcECs) were purified by puromycin selection. These genomic editing cell lines were used in subsequent experiments.

### 4.13 Assay of intracellular ROS

The ROS detection assay was purchased from Beyotime, and was measured with the non‐fluorescent probe 2′,7′‐dichlorofluorescein diacetate (DCFH‐DA). DCFH‐DA easily diffuses into cells and is then deacetylated by esterases to convert into insoluble nonfluorescent DCFH. In the presence of ROS, DCFH reacts with ROS to form the fluorescent product DCF, which is trapped inside the cells. RaECs or other cells and their HDAC1 knockdown cells were seeded at a density of 5 × 10^4^/well in 96‐well plates. One day after seeding, the culture wells were treated with Ang II (2 μM) and DA solution at different doses for 24 h. To obtain dissociated microglia for the ROS assay, the culture medium was first removed and the cells were washed three times with PBS. DCFH‐DA, diluted to a final concentration of 10 μM with serum‐free culture medium, was added to cultures and incubated for 20 min at 37°C. The fluorescence was read at 488 nm with an IN Cell Analyzer 6000 plate reader (Life). The fluorescence intensity indicated intracellular ROS.

### 4.14 RNA‐seq

To better elaborate the role of DA in treating vascular aging, we performed RNA‐seq by dividing RaEC samples into three groups: control, Ang II (2 μM), and Ang II + DA (100 μM). Ang II was used to induce in vitro cellular senescence model as described previously.^51^ The sequencing work was manipulated by Beijing Biomarker Technology (http://www.biomarker.com.cn). GSEA was performed by GSEA 4.3.2 software.

### 4.15 Whole blood co‐culture assay

Whole blood was isolated through the abdominal aorta of rats in a Paxgene blood RNA tube (BD), and the RNA level of aging biomarkers was detected from 1 to 9 h. DA was co‐cultured with whole blood at 50, 100, and 150 μM and p16 and p21 expression was examined at 4 and 8 h. One milliliter of blood was used each time. One milliliter of blood was co‐cultured with ice‐cold red blood cell lysis buffer (Solarbio) for 15 min and then centrifuged at 450 × *g* for 10 min at 4°C. The supernatant was discarded, and the precipitated cells were lysed by TRIzol (Invitrogen).

### 4.16 Statistical analysis

The data are expressed as the mean ± s.d. as indicated. Biological replicates were performed in all experiments unless otherwise stated. One‐way or two‐way analysis of variance was used to analyze the significant differences in the data when more than two groups were compared (multiple comparisons), as indicated. All statistical analyses were performed with GraphPad software 6.0. The threshold for statistical significance was *p* < 0.05.

## AUTHOR CONTRIBUTIONS

Y.Z.Z. and Z.X.L. conceived and designed the experiments. Z.X.L. analyzed the data. Z.X.L., H.H., Y.X., J.H.C., X.Y.L., Q.Y.G., M.H.G., Q.Z., C.K.M., X.Z., and W.Q. performed the validation experiments. Y.M.Z., L.L., and X.Y.Y. helped to improve the grammar. All authors have read and approved the final manuscript.

## CONFLICT OF INTEREST STATEMENT

The authors declare they have no conflicts of interest.

## ETHICS STATEMENT

All animal studies were approved by the Animal Care and Use Committee of the Municipal Affairs Bureau of Macau (approval number AL010/DICV/SIS/2018).

## Data Availability

The data are available from the corresponding author on reasonable request.
